# Factors that support readiness to implement integrated evidence-based practice to increase cancer screening

**DOI:** 10.1186/s43058-022-00347-6

**Published:** 2022-10-06

**Authors:** Cindy Soloe, Laura Arena, Dara Schlueter, Stephanie Melillo, Amy DeGroff, Florence Tangka, Sonja Hoover, Sujha Subramanian

**Affiliations:** 1 RTI International, 3040 E. Cornwallis Road, Durham, NC 27709 USA; 2 Division of Cancer Prevention and Control, National Center for Chronic Disease Prevention and Health Promotion, Centers for Disease Control and Prevention, Atlanta, GA USA

**Keywords:** Colorectal cancer screening, Public health, Partnership, Evidence-based interventions

## Abstract

**Background:**

In 2015, the Centers for Disease Control and Prevention (CDC) funded the Colorectal Cancer Control Program (CRCCP), which partners with health care systems and primary care clinics to increase colorectal cancer (CRC) screening uptake. We interviewed CRCCP stakeholders to explore the factors that support readiness for integrated implementation of evidence-based interventions (EBIs) and supporting activities to promote CRC screening with other screening and chronic disease management activities in primary care clinics.

**Methods:**

Using the Consolidated Framework for Implementation Research (CFIR), we conducted a literature review and identified constructs to guide data collection and analysis. We purposively selected four CRCCP awardees that demonstrated ongoing engagement with clinic partner sites, willingness to collaborate with CDC and other stakeholders, and availability of high-quality data. We gathered background information on the selected program sites and conducted primary data collection interviews with program site staff and partners. We used NVivo QSR 11.0 to systematically pilot-code interview data, achieving a kappa coefficient of 0.8 or higher, then implemented a step-wise process to identify site-specific and cross-cutting emergent themes. We also included screening outcome data in our analysis to examine the impact of integrated cancer screening efforts on screening uptake.

**Results:**

We identified four overarching factors that contribute to clinic readiness to implement integrated EBIs and supporting activities: the funding environment, clinic governance structure, information sharing within clinics, and clinic leadership support. Sites reported supporting clinic partners’ readiness for integrated implementation by providing coordinated funding application processes and braided funding streams and by funding partner organizations to provide technical assistance to support efficient incorporation of EBIs and supporting activities into existing clinic workflows. These actions, in turn, support clinic readiness to integrate the implementation of EBIs and supporting activities that promote CRC screening along with other screening and chronic disease management activities.

**Discussion:**

The selected CRCCP program sites supported clinics’ readiness to integrate CRC EBIs and supporting activities with other screening and chronic disease management activities increasing uptake of CRC screening and improving coordination of patient care.

**Conclusions:**

We identified the factors that support clinic readiness to implement integrated EBIs and supporting activities including flexible funding mechanisms, effective data sharing systems, coordination across clinical staff, and supportive leadership. The findings provide insights into how public health programs and their clinic partners can collectively support integrated implementation to promote efficient, coordinated patient-centered care.

**Supplementary Information:**

The online version contains supplementary material available at 10.1186/s43058-022-00347-6.

Contributions to the literature

- Describes factors that support readiness for integrated implementation of EBIs and supporting activities to promote CRC screening with other screening and chronic disease management activities in primary care clinics
- Provides insights into how public health programs and primary care clinics can collectively support the integrated implementation of interventions within clinic workflows to support efficient, coordinated patient-centered care
- Includes a defining set of constructs derived from implementation research studies that relate to the integrated implementation of chronic disease management interventions and activities within clinical settings

## Introduction

Collaboration between public health and primary care is seen as an opportunity to promote health at the individual and community levels [1–3]. Integrated implementation of patient care interventions and activities can support primary care clinics in improving the efficiency of clinic workflows and coordinating the delivery of patient-centered preventive care [4, 5]. Integrated implementation may also support longer-term outcomes, including clinic institutionalization of health promotion practices and cost savings [6, 7]. Public health partners can play an important role in supporting the implementation of these practices in primary care settings.

In 2015, the Centers for Disease Control and Prevention (CDC) funded the Colorectal Cancer Control Program (CRCCP), which is based on a health systems change model that promotes integrating public health and primary care to improve population health, specifically, to increase colorectal cancer (CRC) screening among people who are underserved by medical services (Table 1) [8].

**Table 1:**
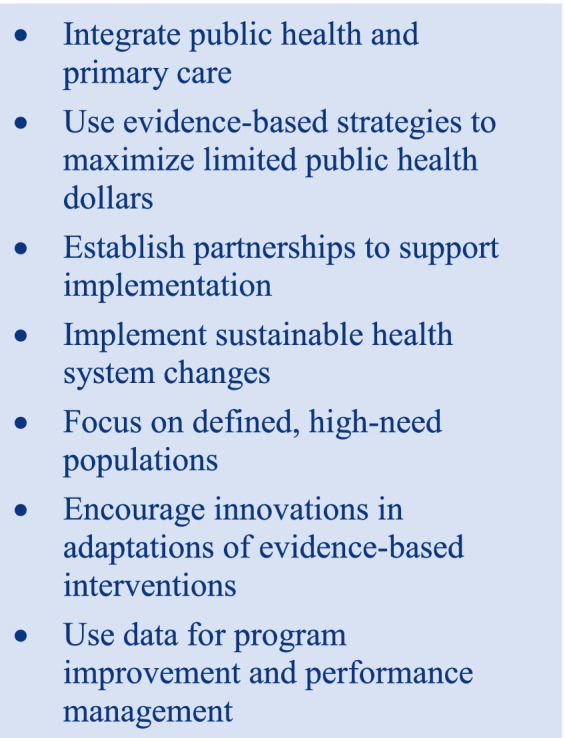
Tenets of the Colorectal Cancer Control Program

CDC funded 30 CRCCP program sites (hereafter referred to as sites) that, in turn, partner with health care systems and their affiliated primary care clinics (hereafter referred to as clinic partners) to support them in implementing evidence-based interventions (EBIs) recommended by the Community Preventive Services Task Force in *The Community Guide* [9] coupled with other supporting activities to increase the uptake of CRC screening. CRC screening and diagnostic tests are reimbursed by payers for those with insurance coverage. The CRCCP DP15-1502 was funded for 5 years from 2015 to 2020. Participating clinic partners selected up to four EBIs recommended by *The Community Guide* including—patient reminders, provider reminders, provider assessment and feedback, and reducing structural barriers to screening—and other supporting activities to implement based on clinic priorities and feasibility. Supporting activities include implementing community health worker-led activities, health information technology, and professional development.

CRCCP sites engage partners with relevant expertise, such as the American Cancer Society and the State Primary Care Association, and together provide technical support to implement these activities. Because the CRCCP is focused on health systems change—such as improving workflows and electronic health record (EHR) systems to incorporate EBIs and supporting activities so that they are sustainable—the opportunity exists to integrate the implementation of EBIs and supporting activities to promote not just CRC screening but also other screening and/or chronic disease management activities. This integrated approach is intended to increase the uptake of screening, improve the coordination of patient care, and improve the efficiency of clinic workflows.

This study aimed to identify factors that support readiness for this integration within CRCCP clinics where EBIs and supporting activities to promote CRC screening were integrated with other screening and chronic disease management activities. The study findings can be used by the CRCCP and other CDC-administered chronic disease programs (e.g., National Breast and Cervical Cancer Early Detection Program (NBCCEDP) and Well-Integrated Screening and Evaluation for WOMen Across the Nation (WISEWOMAN)) as well as other organizations that are planning for or engaged in integrating CRC screening with other health promotion activities.

## Methods

We used the Consolidated Framework for Implementation Research (CFIR) [10] to structure our approach to identify the factors that support readiness to implement integrated EBIs and supporting activities, focusing on characteristics of readiness within the inner setting (clinics, in this context). Using the CFIR, we conducted a literature review to identify constructs derived from implementation research studies that are relevant to integrated implementation and align with the CFIR inner setting construct of interest: readiness for implementation (i.e., available resources, access to knowledge and information, and leadership engagement). Our literature review yielded four key integrated implementation constructs (Table 2) that align with these CFIR constructs. Together, these theory-based constructs provided a strong foundation for the areas of implementation on which to focus and guided data collection and analysis efforts.

**Table 2:** Guiding Consolidated Framework for Implementation Research (CFIR) and integrated implementation construct descriptions

**CFIR construct and definition**	**Integrated implementation construct**	**Integrated implementation construct description**
**Readiness for implementation: available resources** ***The level of resources dedicated for implementation and ongoing operations, including money, training, education, physical space, and time***	Funding environment	Integrated health and social services are supported by financing mechanisms that fund services and allow braiding or blending of funds with flexibility in the use of funds to achieve population health goals [2, 11–14]. Integrated services also may be supported by partner agencies that receive funding to provide training and technical assistance to assist clinics to adapt clinical workflows.
**Readiness for implementation: access to knowledge and information** ***Ease of access to digestible information and knowledge about the intervention and how to incorporate it into work tasks***	Governance structure	Cooperation between and within organizations to support integrated health care delivery requires governance structures that promote coordination, joint planning, shared priorities, and a common understanding of accountability for patient care among staff [11, 15–17].
	Information sharing	Information flow in a clinical setting is necessary for integrated health care delivery and is supported by the presence of a secure, accessible platform for storing and sharing health care information, consistent documentation, and a structured plan to facilitate seamless communication among health system care providers [11, 13, 18].
**Readiness for implementation: leadership engagement** ***Commitment, involvement, and accountability of leaders and managers with the implementation***	Leadership support	Leadership recognition of the importance of integration and provision of tangible support and resources are influential in the adoption and implementation of care integration [19–24].

**Fig. 1:**
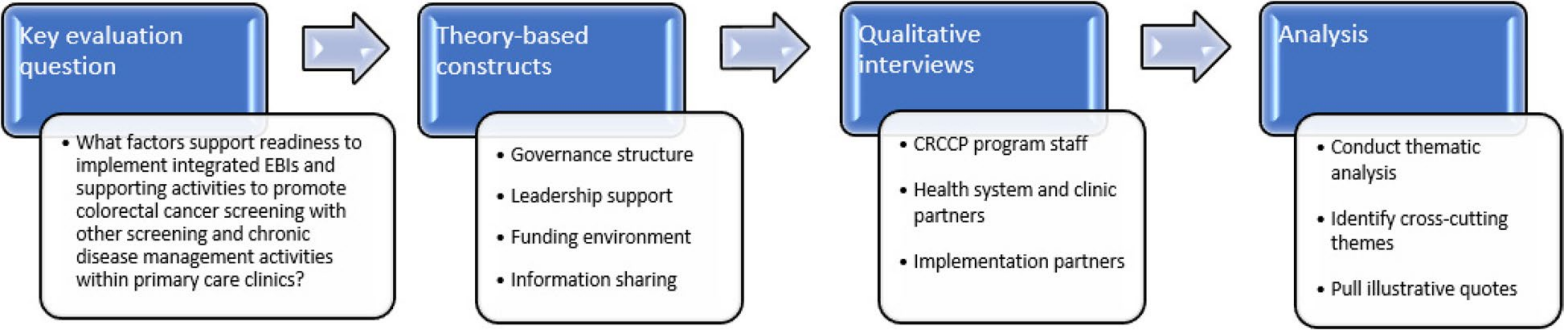
Methodological approach

Our primary evaluation question was, “What factors support readiness to integrate implementation of EBIs and supporting activities to promote CRC screening with other screening and chronic disease management activities within primary care clinics?” We developed additional evaluation questions (see Additional file 1) related to each of the key constructs in Table 2. An overview of our methodological approach is provided in Fig. 1, and further detail about site selection, data collection, and analysis are provided below.

### Site selection

We purposively selected three CRCCP sites with clinic partners who were integrating the implementation of CRC EBIs and supporting activities (see Table 3). These sites included the Kentucky Department of Public Health, the Rhode Island Department of Health, and the Washington State Department of Health. We also included the Nebraska Department of Health and Human Service’s CRC program, which was a CRCCP awardee from 2009 to 2015. Although Nebraska was not a CRCCP awardee at the time of the study, they maintained program efforts with state funds throughout our study period. These four sites participated in the CRCCP Learning Collaborative, an initiative to develop and apply a standardized approach to evaluate the implementation, effectiveness, cost, and cost-effectiveness of multicomponent interventions to inform the future scale-up of these interventions [7]. Based on cost-effectiveness analyses, all four sites exhibited partnerships with clinics to integrate the implementation of CRC EBIs and supporting activities.

**Table 3:** Brief description of clinic partner integrated implementation approaches, by site

**Site**	**Approaches to integrated implementation by clinic partners**
**Rhode Island**	Used a chronic care delivery model that integrates CRC screening with other cancer and chronic disease screenings. Integration of CRC with other chronic disease screenings is reflected in workforce development, clinical practice guidelines, EHR prompts with physician reminders, and patient navigation
**Nebraska**	Integrated CRC screening within an EHR-based provider reminders system (clinical decision support rules) used for multiple screenings Used a FluFIT program to integrate CRC screening (FOBT kits or colonoscopy referral) with flu shots
**Washington**	Integrated CRC screening within EHR-based patient and provider reminders that are used for multiple screenings Expanded interventions focused on reducing structural barriers (e.g., providing mobile mammography and transportation vouchers) to include barriers to CRC screening (e.g., mailing FIT kits to patients due for CRC screening)
**Kentucky**	Integrated CRC screening into an existing patient reminder system (i.e., phone calls to remind patients about the need for CRC screening, other cancer screenings). Reduced structural barriers for CRC screening by including screening as part of “max packing” appointments that also included flu shots and/or mammograms

*Note*: *CRC* colorectal cancer, *EHR* electronic health record, *FIT* fecal immunochemical test, *FOBT* fecal occult blood test

### Data collection

For each selected program, we interviewed three participant types—staff, clinic partners, and implementation partner organizations (e.g., non-clinic partners funded to provide technical assistance to clinics)—and collected screening uptake data for each partner clinic.

For interviews, we used the four theory-based integrated implementation constructs—governance structure, information sharing, funding environment, and leadership support—and evaluation questions to inform the development of unique interview guides for each of the three participant roles. We selected these participant types to gather input from multiple perspectives. Sample interview questions are provided in Additional file 2. To gather contextual information about each program prior to conducting interviews, we reviewed the secondary data when available (e.g., budgets and survey findings by program)^1^ and spoke with the CDC staff who provided tailored technical assistance to the three selected sites.

We obtained verbal consent for interviews from each participant. Institutional Review Board approval was not required for this data collection because it did not constitute human subject research. Office of Management and Budget approval was not required given that no more than nine respondents answered each set of questions in the unique interview guides. In total, 23 individuals, including representatives from one clinic partner location per program, participated in individual semi-structured telephone interviews (Table 4) conducted between February and May 2019.^2^ Interviews were audio-recorded with consent, and the recordings were transcribed for analysis.

**Table 4:** Interviews by program site^a^ and respondent type and site

**State**	**Program staff**	**Clinic partner staff**	**Implementation partner staff**	**Total interviewees**
**Kentucky**	2	2	2	6
**Nebraska**	2	2	2	6
**Rhode Island**	2	2	2	6
**Washington**	2	1	2	5
**Total**				23

^a^Kentucky Department of Public Health, Nebraska Department of Health and Human Services, Rhode Island Department of Health, and Washington State Department of Health

We also requested breast, cervical, and CRC screening uptake data from the CDC for each of the sites for 2017–2019. CDC collects clinic-level data from all participating clinics annually, including screening uptake, which includes screening mammography, pap test, pap/HPV test, colonoscopy, FIT, FOBT, and flexibility sigmoidoscopy, depending on what is offered or referred by the clinic [25]. We examined these data after our interviews to explore the changes in breast, cervical, and CRC screening uptake during the 3-year period of integrated cancer screening program implementation (2017–2019). Across all sites, we refer to each 1-year period as a program year.

### Analysis

Prior to qualitative analysis, we developed a coding dictionary based on our evaluation questions (see Additional file 3). A team of four analysts pilot-coded two interview transcripts using the qualitative software NVivo QSR 11.0. The team then met to develop a consensus regarding the refinement and application of the coding framework. Four interviews (20%) were double coded—independently coded by two analysts—and analysts achieved a kappa coefficient of 0.8 or higher for each, indicating excellent inter-rater reliability. The remaining interviews were divided evenly among the four analysts and independently coded. Following coding, analysts independently reviewed the code reports to identify program-specific emerging themes and recorded these themes in summary tables that included a description of each theme, illustrative quotes supporting the theme, and the interviewee’s role for each quote. Analysts convened and reviewed all theme tables and identified cross-cutting emergent themes, which were themes that emerged across at least two programs. Analysts reviewed and refined cross-cutting themes until reaching an agreement. We calculated the percentage point changes from program year 1 to program year 3 in breast, cervical, and CRC screening uptake by clinic and reported (by site) whether screening rates increased, decreased, or remained the same.

## Results

**Fig. 2:**
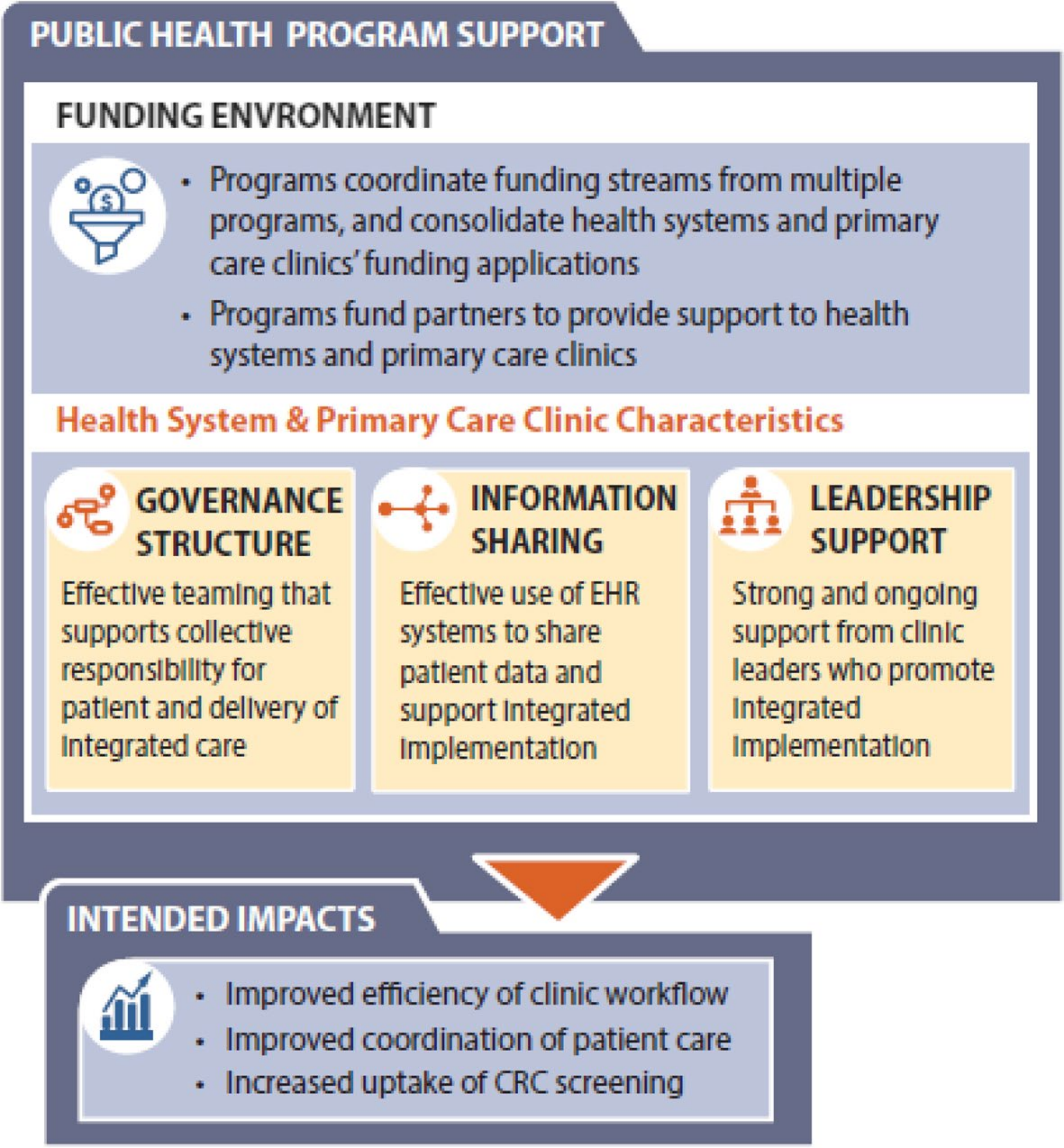
Program site and clinic partner factors supporting readiness for integrated implementation

In the following sections, we present our findings on the factors that support readiness for integrated implementation. These factors are summarized in Fig. 2.

### Funding environment

The clinic partners received funding from the CRCCP sites, typically to support start-up and ongoing costs associated with implementing the CRCCP within their clinics. Two aspects of the funding environment were identified as facilitating readiness for integrated implementation: (1) coordinating funding opportunities across multiple chronic disease programs to support consolidated application processes for clinic partners and (2) contracting with expert implementation partners to provide training and technical assistance to clinic partners that emphasized integrated implementation.

Participants discussed programs providing braided funding—a process that involves coordinating separate funding streams from multiple programs, such as CRCCP and NBCCEDP—to pay for common activities such as patient navigation across programs, provider reminders, and patient reminders (e.g., reminders for breast, cervical, and CRC screening). However, each funding stream is kept separate so programs can track requirements and outcomes. For example, the program site staff discussed developing funding opportunity announcements for clinic partners that braided funding streams from multiple programs. This approach enabled clinic partners to consolidate their funding applications and reporting processes while tracking distinct activities and outcomes for each funding stream. Participants also reported that by braiding funding from multiple chronic disease programs, clinic partners could submit a single funding application and receive a larger amount of funding that could be used to integrate implementation efforts across multiple chronic diseases. For instance, participants reported the use of braided funding to support patient navigation staff who coordinate screening and follow-up for breast, cervical, and CRC.

The [health department’s] women’s cancer screening and colorectal had patient navigation contracts with all the [Federally Qualified Health Centers] throughout the state and in order to get the FQHCs to agree to do the colorectal, right from the beginning she integrated the contracts so we essentially were doubling the money that we were offering to them and it was an all or nothing kind of thing. – Program site staff

[We aim to] present different contract options which combine all the different funding sources…in an integrated way, approaching them with this single menu of different options collectively…versus one of us [from the health department] approaching them one month and then another one approaching them 3 months later. *‒* Program site staff

[Braided funding supports] staff time and the training that we need for our staff to do the outreach for all of the cancer screenings. – Clinic staff

Participants also described how technical assistance and training, provided by expert implementation partners, facilitated integrated implementation. For example, implementation partners assisted clinics in adapting EHR or other referral systems to integrate CRC screening with existing referral systems for breast cancer screening such as mammography.

[Implementation partner agency] is our partner in understanding how to look at [clinic] practice flows and how to coordinate, integrate, align the work we do…. They say, ‘Okay, you really need to work on your electronic referrals [for screening]. Let’s look at how you’re doing that with mammograms. Let’s look at how you’re doing that with colonoscopy.’ *–* Program site staff

We strongly encourage [clinic partners] to consider how their efforts could be better integrated with their other programs and activities in their clinic systems. For example, when they describe [clinic] workflows to us on these technical assistance calls, we try to prompt them to consider how these efforts may impact other efforts ongoing in their clinics, other screening activities…if they’re going to look at, for example, whether or not a patient is due for colorectal cancer [screening as part of workflow processes], seeing if there are opportunities in their other cancer screening activities and workflows. *–* Implementation partner staff

### Governance structure

Having the proper governance structure can lead to shared priorities and a sense of collective responsibility for patient care among clinic staff. Participants described effective team-based care, a factor related to governance structure, as important in ensuring that CRC screening was integrated into clinic practice as part of a comprehensive, coordinated approach to patient care. For example, participants described training all clinic staff—such as patient registration staff, lab technicians, and nurses—so that they are all able to effectively address CRC and other health conditions with each patient encounter. Additionally, participants described the integration of CRC screening with other screening and chronic disease management activities as being consistent with their commitment to applying a Patient-Centered Medical Home (PCMH) model.

Going back to the PCMH [model] of having all staff [perform at] the highest ability that they’re able to. So, our frontline, our medical assistants, our front-desk staff are very involved and very engaged in these management activities, cancer screening activities. – Clinic staff

Every clinical person from our lab person to our [patient] registration staff to every nurse [and] medical assistant understands that they’re required to address all of these [health topics] with every patient. It’s just the way we train them when they come in…it’s the way we do business. – Clinic staff

We have a team medical assistant [who] is able to follow up on closing the loop to patients about, ‘Hey, I see you haven’t done this,’ or ‘Your A1c was high. We need you to come back in or follow up on those things.’ And then the RN is able to really take the time and educate the patients on different dietary concerns, different ways to manage whatever specific chronic condition that they have. – Clinic staff

### Information sharing

The ability to access and share accurate patient information, including EHR data, was identified as another factor supporting readiness for integrated implementation of CRC screening. Identifying patients who are due for multiple preventive screenings, including CRC, was an example shared by participants. Once patients could be identified, referrals could be made and follow-up actions—such as appointment scheduling and confirming screening completion—could be carried out.

Participants indicated that the clinic staff, particularly patient navigators and care coordinators, rely on the availability of accurate EHR reports to identify patients for screening and/or diagnostics for multiple chronic disease conditions. The clinic staff emphasized that the utility of the EHR data in supporting integrated implementation is contingent on data accuracy.

The challenges we face with colorectal cancer, breast cancer, and cervical reporting [are] the same challenges we face with everything else. Making sure [EHR data] are entered correctly and data validation. – Clinic staff

Aside from EHRs, participants identified the use of data dashboards and holding quality improvement team meetings as strategies for sharing information that facilitated integrated implementation. Electronic dashboards presented summary metrics on multiple cancer screenings in real time for each provider and their respective patients. Through data sharing and making comparisons between physicians that invite friendly competition, the dashboards promote action on multiple conditions that contribute to integrated implementation. Similarly, data sharing among quality improvement (QI) teams promotes a collective understanding of where clinics stand on the delivery of health promotion activities that can foster understanding of opportunities to potentially improve these metrics through integrated implementation.

All of our staff have access to the provider dashboard, which is updated once a month and that shows where their particular provider is and what the [clinic] average is and then… they can go and look at any other provider…it’s just starting with the cancer screening metric, but eventually we’ll put all of our metrics on that…it will give them more of a real-time feel of where they’re at. – Clinic staff

We also…had monthly quality improvement meetings, where all of the clinic’s leadership and the QI department got together, and we talked about things that we are working on, and things that could potentially be shared…beyond just cancer, or beyond just diabetes care, or whatever thing we were talking about. – Clinic staff

### Leadership support

Participants described leadership as playing an important role in supporting clinic readiness for integrated implementation by promoting expectations for the uptake of integration practices. Participants indicated that strong and ongoing support from the health system and clinic leadership for integrating CRC EBIs and supporting activities within the clinic practice and incorporating CRC screening efforts with other cancer or chronic disease management activities set the stage for clinic receptivity or readiness. For example, participants noted that leaders need to talk about the importance of implementation and integration of EBIs and supporting activities in order to set the tone that these activities are important and subsequently engage staff in uptake.

I’m the clinic manager and so I set the tone for the sense that the evidence-based interventions are important. We need to integrate them and so you talk about it, you bring it up frequently. As a group, we collectively talk about what we think works for us and what doesn’t work for us. ‒ Clinic staff

Leadership support is critical.… It’s evident, if you see the [screening] numbers of the teams that have the leadership support and the ones from the team that didn’t [have leadership support], it’s night and day. ‒ Implementation partner staff

### Intended impact of integrated screening implementation

Interview participants shared insights into the intended impact of integrated implementation including increased uptake of CRC screening, improved coordination of patient care, and improved clinic efficiency in terms of both cost and time.

We have seen improvements in our colorectal cancer screening rates…in all of our uniform data set information and quality indicators … I really do, I think it just comes down to the integrated approach. – Clinic staff

I think integrated implementation, regardless of which EBI you pick promotes accountability for providers and their teams to make sure that they are providing the best care for the whole person. – Clinic staff

I think the biggest benefit that I can see is that it’s really cost saving and time savings... these EBIs are cross-cutting across all of these cancer screenings. – Clinic staff

I can’t even imagine not integrating certain components of programs… it really helps us to get things done much faster. – Grantee

### Screening uptake

In Table 5, we show the percentage point changes from program year 1 to program year 3 in breast, cervical, and CRC screening uptake by site. All sites showed increases in breast cancer screening uptake ranging from 1 percentage point in Washington1 to 22.1 percentage points in Nebraska1. The results were mixed for cervical cancer, as two sites showed increases in cervical cancer screening uptake, two showed decreases, and two showed no change at all. There were increases in CRC screening uptake at four sites ranging from 6.4 percentage points to 14.1 percentage points and decreases at two sites (5.9 and 12.1 percentage points).

**Table 5:**
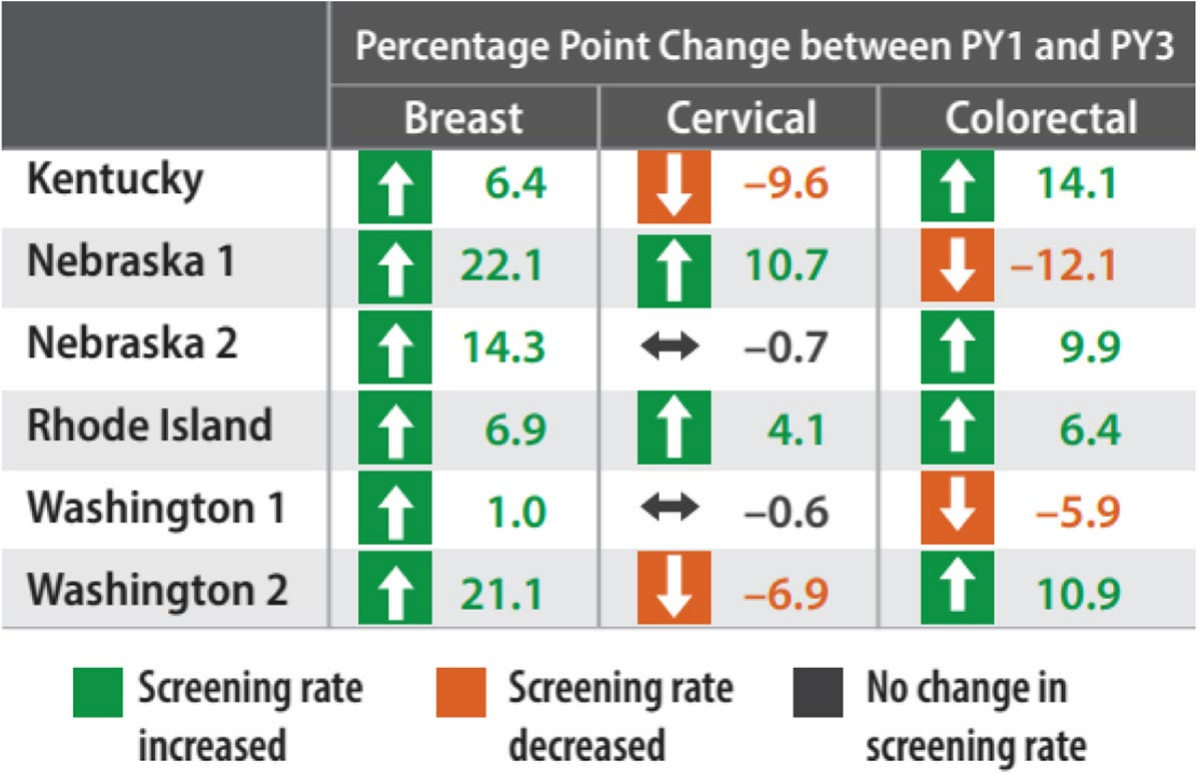
Changes in breast, cervical, and colorectal screening uptake by site from program year 1 to program year 3

Source: Breast and cervical cancer screening data are from the Centers for Disease Control & Prevention’s National Breast and Cervical Cancer Early Detection Program; Kentucky, Rhode Island, and Washington CRC screening data are from the CDC’s Colorectal Cancer Control Program. Nebraska CRC screening data are from the Nebraska Department of Health and Human Service

## Discussion

Although readiness to adopt evidence-based interventions and integrated health care delivery models has been explored in depth [12, 26–36], few studies have explored clinic readiness to adopt integrated delivery of cancer screening and chronic disease management activities in the USA. Our study examined the CRCCP, a public health program implemented in primary care clinics, to identify the factors that support readiness for integrated implementation of CRC EBIs and supporting activities with other screening and chronic disease management activities. Using the CFIR helped focus the identification of factors that support readiness for integrated implementation of EBIs, including funding environment, governance, information sharing, and leadership.

We found that readiness for integration in clinics was supported when programs consolidated funding into single contracts for partner clinics, rather than providing separate “siloed” contracts for individual health conditions. In the CRCCP, this approach presents an opportunity for more efficient use of public health funding for coordinated promotion of cancer screenings (e.g., colorectal, breast, and/or cervical). Efficiencies are also achieved when the clinic staff respond to a single, consolidated funding application rather than multiple applications, and the pooled funding provides both program sites and clinic flexibility to leverage funds and share overhead costs, offering the opportunity to achieve more with their awarded funds [37]. At the program site level, consolidated funding may also support the delivery of more integrated training and technical assistance to awarded program sites, meaning more comprehensive support could be provided to trainees.

At the clinic level, a team-based approach was found to support staff ability to integrate implementation of EBIs and supporting activities to promote CRC screening with other patient care activities. Teams with effective communication strategies were best positioned to align CRC screening efforts efficiently into existing clinic workflows (e.g., identifying patients who are due for CRC screening and determining how best to integrate this information within existing provider reminder practices). This integration of information flow within clinics reflects a patient-centered approach to care, an approach that can ultimately lead to improved patient outcomes and quality of life [38, 39] as well as reduced health care costs [40].

EHR systems, a reservoir of patient information, were also found to be an important component of information sharing to support integrated implementation. Although investing in functional EHR systems can be costly, these systems are recognized as essential to enable optimal, integrated, patient-centered care because they allow for the abstraction of accurate clinical information. In 2011, the Centers for Medicare & Medicaid Services established an EHR incentive program to reward meaningful use of certified EHR systems to improve the quality of care [41]. Since then, there has been a national push for health systems to adopt EHRs to support clinical practice transformation. The meaningful use of EHRs has been associated with quality improvement in Federally Qualified Health Centers (FQHCs) [42]. In 2012, 90% of FQHCs had adopted an EHR, and about one-third had met the core requirements for meaningful use [43]. The findings from our study indicate how EHR enhancements to support CRC EBI implementation can support readiness for integrated implementation to promote other cancer and chronic disease screenings. For example, once developed, data dashboards that display provider performance metrics for CRC screening can be replicated or expanded to produce monitoring data for other cancer or chronic disease screenings, placing patients at the center of care provided by multiple public health programs. Additionally, EBIs that can be enabled through the EHR system, such as automated patient and provider reminders or performance metrics to populate dashboards, may be more sustainable. Once these strategies are built into the EHR systems, significant efficiencies can be achieved.

Finally, leaders’ expectations for integrated implementation can set the tone for clinic staff, creating a culture of readiness for the uptake of integration. For example, leadership support of CRC screening champions, individuals serving as internal advocates for screening, can help add credibility to champion activities. This is important as clinic champions have been found to contribute to improved public health outcomes in many areas [44]. Furthermore, champion efforts have been associated with screening rate increases in CRCCP clinics [8]. As such, leadership support of champions and their efforts to sustain and support integrated implementation as part of the CRCCP is crucial.

Our findings highlight elements and practices of integrated implementation that can support clinics in achieving desired short-term outcomes, including efficient, coordinated delivery of interventions to ensure patient-centered care, which can ultimately lead to improved patient outcomes as well as reduced health care costs [38, 39]. Our results show that screening uptake generally increased across all three types of cancer screenings, breast, cervical, and CRC, for these sites with integrated screening promotion activities. Some of the decreases in screening rates were likely influenced by other external factors. For example, Nebraska reported a general decrease in CRC screening because of floods that devastated the state and impeded clinic capacity to engage patients during the timeframe of this study. Furthermore, it is likely that contextual factors, such as serving patients with complex care needs (e.g., comorbidities) and the impact of social determinants of health (SDOH), merit consideration in EBI planning and integrated implementation. Future studies might explore more directly the impact of SDOH and other contextual factors on the interplay between integrated implementation and screening uptake.

CDC, through the CRCCP Learning Collaborative, is working with program sites and clinic partners to systematically evaluate the cost and cost-effectiveness of integrated implementation of EBIs for cancer screenings [7]. Lessons learned through the integrated implementation of cancer screenings through the CRCCP might be applied to enhance other public health and primary care partnership programs, such as CDC’s NBCCEDP or WISEWOMAN. For example, findings might be applied to inform the structure of public health department partnerships with clinic and non-clinic partners, inform the nature of training and technical assistance provided to primary care clinics, encourage early efforts to seek clinic leadership buy-in to setting the stage for integration, and reiterate the value of capturing high-quality data and sharing this information among clinic team staff to support integrated programs.

Our findings should be considered within the context of certain limitations. Because the study was qualitative and engaged a subset of current and former CRCCP programs and their primary care clinic partners, these findings are not generalizable to all CRCCP programs or primary care clinic sites. The findings should also be considered within the context of the relatively small number of participants included in the data collection. In considering the changes to cancer screening rates (Table 3), it should be noted that the intensity of delivery of screening promotion programs across sites was not captured within the scope of our study, which focused on contextual factors influencing the integration of EBIs and supporting activities to promote CRC screening with other clinic activities. Additionally, this study was framed around the exploration of four theory-driven constructs. Future analysis that incorporates a broader set of constructs may generate additional insights regarding factors that support the integrated implementation of interventions for multiple conditions within primary care. Finally, we did not track the outcomes for the specific time period reflected in this qualitative analysis but plan to engage in such assessments in the future.

## Conclusion

In this study, we identify the factors within CRCCP primary care clinics that support readiness for integrated implementation of EBIs and supporting activities to promote CRC screening with other screening and chronic disease management activities. Our findings yielded four strategies that can support readiness to implement integrated screening promotion within the inner setting of primary care clinics: (1) funders may consider flexible funding streams so health systems can blend funds and use them efficiently, (2) primary care clinics may consider training a broad range of providers on integrated screening promotion so a team-based approach can be employed to support patients at all touch points, (3) health system and clinic leadership support must be present or developed for successful adoption, implementation, and maintenance of integrated screening promotion efforts, and (4) health systems may consider investing in systems and tools to allow for sharing patient data so multiple screening needs can be monitored and addressed in a coordinated manner. Future studies could include a comprehensive assessment of the barriers, facilitators, and impact of the integrated implementation of EBIs and supporting activities to promote CRC screening and other cancer screening and chronic disease management activities on patient and program outcomes.

## Supplementary Information

**Additional file 1. **Primary Evaluation Questions. This document presents additional evaluation questions related to each of the key constructs in Table 2.

**Additional file 2. **Sample Interview Questions (Table of sample questions related to each construct). This document presents sample questions for the qualitative interviews.

**Additional file 3. **Coding Structure (23 Codes developed based on evaluation questions). This document provides a coding dictionary based on the evaluation questions.

## Data Availability

The qualitative data generated and/or analyzed during the current study are not publicly available because they were generated in interviews conducted by the research team, with the expectation that participant identity would be kept confidential.

## Abbreviations

CDC: Centers for Disease Control and Prevention
CFIR: Consolidated Framework for Implementation Research
CRC: Colorectal cancer
CRCCP: Colorectal Cancer Control Program
EBIs: Evidence-based interventions
EHR: Electronic health record
FQHC: Federally Qualified Health Center
PCMH: Patient-Centered Medical Home

## Footnotes

1 Findings were drawn from an annual survey administered by CDC to assess programs’ partnerships, technical assistance provided to clinics (e.g., data quality improvement), and technical assistance needs.

2 One interview included two clinic partner staff.
