# Supplementary material for: Factors that support readiness to implement integrated evidence-based practice to increase cancer screening
Source: Implement Sci Commun. 2022 Oct 6;3:106. doi: 10.1186/s43058-022-00347-6 (PMC9535984; doi:10.1186/s43058-022-00347-6)
Supplement: Supplementary file 1 — Additional file 1. Primary Evaluation Questions. This document presents additional evaluation questions related to each of the key constructs in Table 2. [file 43058_2022_347_MOESM1_ESM.docx]

**Additional file 1.** Primary evaluation questions

|  | Primary Evaluation Questions |
| --- | --- |
| Governance structure | - How are programs and clinics structured to support integrated implementation of EBIs and supporting activities? - How are health teams being used as part of integrated implementation of EBIs and supporting activities? - What does integrated implementation of EBIs and supporting activities look like in terms of clinic workflow? - What activities are conducted at the clinic to facilitate integration of EBIs and supporting activities implementation with clinic processes? |
| Leadership support | - How is clinic leadership supporting integrated implementation of EBIs and supporting activities? - How is program leadership supporting integration? |
| Funding environment | - How are multiple lines of funding (e.g., CRC, NBCCEDP) to clinics coordinated to support integrated implementation of EBIs and supporting activities? - To what extent are the programs and/or their partners (clinic and implementation partners) sharing resources (monetary and other) to support integrated implementation of EBIs and supporting activities? - In what ways do programs incentivize or encourage integrated implementation of EBIs and supporting activities at the clinic level? |
| Information sharing | - How is information shared within clinics and health systems to support integrated implementation of EBIs and supporting activities? - What challenges and benefits have clinics encountered in coordinating data entry and reporting around integrated implementation of EBIs and supporting activities? - How does a clinic’s EHR system facilitate/hinder efforts to integrate implementation of EBIs/supporting activities? |

Note: NBCCEDP, National Breast and Cervical Cancer Early Detection Program; CRC, colorectal cancer; EBI, evidence-based interventions; EHR, electronic health record.
