# Supplementary material for: Factors that support readiness to implement integrated evidence-based practice to increase cancer screening
Source: Implement Sci Commun. 2022 Oct 6;3:106. doi: 10.1186/s43058-022-00347-6 (PMC9535984; doi:10.1186/s43058-022-00347-6)
Supplement: Supplementary file 2 — Additional file 2. Sample Interview Questions (Table of sample questions related to each construct). This document presents sample questions for the qualitative interviews. [file 43058_2022_347_MOESM2_ESM.docx]

**Additional file 2.** Sample interview questions (table of sample questions related to each construct)

| Construct | Sample Interview Questions |
| --- | --- |
| Governance structure | - In what ways does staffing structure at the health department support integrated implementation of CRCCP activities with those of other cancer or chronic disease area programs? - In what ways does staffing structure at the clinic support implementation of EBIs across multiple cancer or chronic disease area programs? - To what extent is integrated implementation reflected in clinic standard operating procedures? - Based on your experience, what do programs or clinics need in terms of infrastructure to be able to successfully integrate implementation of EBIs? |
| Leadership support | - In what ways does your health department leadership support integration of CRCCP with other cancer or chronic disease area programs? - In what ways does your health department leadership support integrated implementation of EBIs at the clinic level? - In what ways does the health department support integrated implementation of EBIs at your clinic? - In what ways is clinic leadership supporting integrated implementation of EBIs? |
| Funding environment | - To what extent are multiple lines of funding (e.g., colon cancer, breast, cervical, heart disease, and diabetes screenings) to clinic sites coordinated to support integrated implementation of EBIs? - In what ways does the health department incentivize or encourage integrated implementation at the clinic level? - How have you used funding from the health department to support integrated implementation of EBIs? - What influence do policies and/or incentives (national quality incentive programs) have on improvement of integrated implementation of EBIs? - Does the health department provide your organization with funding for your role in supporting integrated implementation of EBIs in their clinic partners? |
| Information sharing | - How is programmatic information, such as clinical or financial information, shared between your program and other chronic disease programs with which you’re collaborating at the health department? - How is information shared within clinics and health systems to support integrated implementation of EBIs? - Thinking about the logistics of documentation—such as reporting requirements to the health department, HRSA, your health system, or other entities—have there been any challenges in terms of documentation or data reporting due to integrated implementation of EBIs? Any benefits? |

Note: CRCCP, Colorectal Cancer Control Program; EBIs, evidence-based interventions; HSRA, Health Services Research Administration.
