# Supplementary material for: Factors that support readiness to implement integrated evidence-based practice to increase cancer screening
Source: Implement Sci Commun. 2022 Oct 6;3:106. doi: 10.1186/s43058-022-00347-6 (PMC9535984; doi:10.1186/s43058-022-00347-6)
Supplement: Supplementary file 3 — Additional file 3. Coding Structure (23 Codes developed based on evaluation questions). This document provides a coding dictionary based on the evaluation questions. [file 43058_2022_347_MOESM3_ESM.docx]

**Additional file 3.** Coding structure (23 codes developed based on evaluation questions)

| Construct | Codes |
| --- | --- |
| Implementation | - **Program Role:** How programs (i.e., funded health departments) interact with clinic sites and/or non-health system partners, and how programs support integrated implementation in clinics. - **Partner Role:** How non-health system partners interact with programs and/or clinic sites, length of relationship with the program, and how the partner organization supports the program’s efforts to encourage integration in clinics. - **Program Integration Description:** How programs are integrating CRCCP with other cancer or chronic disease area programs within the health department. - **Clinic Integration Description:** How clinic sites are integrating implementation of colorectal cancer EBIs with breast, cervical, heart disease, diabetes, and/or other screenings within the clinic. - **Integrated Implementation Challenges and Solutions:** Challenges related to integrating cancer and chronic disease prevention programs at the health department and strategies for overcoming challenges. - **Integrated Implementation Facilitators:** Factors that facilitated integrating cancer and chronic disease programs at the health department. - **Integrated Implementation Benefits:** Benefits of integrating implementation of EBIs. - **Lessons Learned/Recommendations for Programs:** Lessons learned or recommendations for other health departments that want to move toward or sustain integration of cancer and other chronic disease programs. - **Lessons Learned/Recommendations for Clinics:** Lessons learned or recommendations for clinics that want to move toward integrated implementation of EBIs to promote cancer and other chronic disease screenings. |
| Governance structure | - **Program Staffing and Infrastructure:** Discussion of health department staffing structure and how it supports integrated implementation of CRCCP activities with those of other cancer or chronic disease prevention programs. - **Clinic Staffing and Infrastructure:** Clinic staffing structure and how it supports integrated implementation of EBIs to promote cancer and chronic disease screenings. |
| Leadership support | - **Program Leadership Support:** How health department leadership supports integration of CRCCP with other cancer or chronic disease prevention programs. - **Clinic Leadership Support:** How clinic leadership supports integrated implementation of EBIs to promote cancer and other chronic disease screenings. |
| Funding environment | - **Coordination of Funding Streams:** How the health department coordinates funding from various sources to support integrated implementation of EBIs in clinics. - **Internal/External Incentives for Integrated Implementation:** Any incentives that the health department provides to clinics to encourage integrated implementation. How external policies and incentives (e.g., National Quality Incentive Programs) affect integrated implementation and/or improvement of integrated implementation of EBIs in clinics. |
| Information sharing | - **Program Information Sharing:** How health department staff share information between CRCCP and other cancer/chronic disease prevention programs. - **Clinic Information Sharing:** How staff within clinics/health systems share information. - **Clinic Reporting:** Any reporting challenges and/or benefits that are a result of integrating implementation of EBIs in clinics. |
| Sustainability | - **Sustainability Plans:** Clinic systems, infrastructure, and/or policies/procedures that are in place to sustain integrated implementation of EBIs. - **Sustainability Challenges:** Challenges related to sustaining integration of cancer and chronic disease prevention programs at the health department. - **Clinic Champions:** The role of champions in supporting integrated implementation of EBIs in clinics. - **Role of Funding:** Resources available to sustain integrated implementation of EBIs in clinics without CDC funding. - **Program Improvement:** Systems, efforts, or processes that support improvement of integrated implementation of EBIs in clinics. |

Note: CDC, Centers for Disease Control and Prevention; CRC, colorectal cancer; CRCCP, Colorectal Cancer Control Program; EBIs, evidence-based interventions.
